# Label-Free Urinary Proteomics Uncovers Immune-Related Non-Invasive Biomarkers for Primary Biliary Cholangitis

**DOI:** 10.3390/ijms27177584

**Published:** 2026-08-24

**Authors:** Xiong Pei, Ting Lei, Wei Jiang, Qingmin Zeng, Hong Tang, Taoyou Zhou, Dongbo Wu

**Affiliations:** Center of Infectious Diseases, West China Hospital, Sichuan University, Chengdu 610093, China; peixion@163.com (X.P.); leiting0624@163.com (T.L.); jiangwei8171@wchscu.cn (W.J.);

**Keywords:** urinary proteomics, primary biliary cholangitis, osteopontin, receptor activity-modifying protein 3, S100A8, non-invasive biomarker

## Abstract

Diagnosis of primary biliary cholangitis (PBC) currently depends on invasive liver biopsy or serum markers with inadequate diagnostic performance. This study aimed to identify non-invasive urinary protein biomarkers for PBC detection. Urine specimens from biopsy-verified PBC patients and healthy controls were processed through ultracentrifugation-based protein extraction, enzymatic digestion, and HPLC-ESI-IT/MS proteomic profiling; protein quantification was completed using Spectronaut v14.8. We identified 194 differentially expressed urinary proteins (109 upregulated, 85 downregulated) and screened 10 immune-related candidates through GO and KEGG enrichment. Pearson correlation further filtered three core proteins, osteopontin (SPP1/OPN), RAMP3 and S100A8, that correlated significantly with key PBC biochemical indices (ALP, GGT, AST, ALT, IgM, *p* < 0.05). Elevated urinary concentrations of OPN, RAMP3 and S100A8 were validated by ELISA in an independent cohort containing 30 PBC patients and 20 healthy volunteers. In summary, urinary OPN, RAMP3 and S100A8 are markedly increased in PBC patients and hold promise as non-invasive diagnostic biomarkers for PBC; however, their diagnostic specificity against other cholestatic and autoimmune liver diseases remains to be evaluated, and further confirmation in larger multicenter cohorts with disease control groups is warranted.

## 1. Introduction

Primary biliary cholangitis (PBC) is a chronic, progressive, autoimmune cholestatic liver disease characterized by non-suppurative destructive cholangitis of small- and medium-sized intrahepatic bile ducts, which may gradually progress to cirrhosis and liver failure [1]. PBC predominantly affects middle-aged women, with a female-to-male ratio of approximately 9:1, although recent epidemiological data suggest an increasing incidence in men and higher associated complication rates and mortality in male patients [2,3]. The global incidence and prevalence of PBC have shown a rising trend over recent decades, with significant geographic variability [3,4].

The pathogenesis of PBC is multifactorial, involving complex interactions between genetic susceptibility, environmental triggers, and dysregulation of both innate and adaptive immune responses [5]. Clinically, approximately 50% of patients are asymptomatic at diagnosis, presenting only with biochemical abnormalities [6]. Without timely intervention, the disease can progress to liver failure within 10–20 years [1]. Therefore, early diagnosis is paramount for improving long-term outcomes. Current diagnostic criteria for PBC are based on biochemical evidence of cholestasis, presence of serum antimitochondrial antibodies (AMA) or AMA-M2 or liver histopathology consistent with PBC [7]. However, each criterion has limitations. GGT levels are susceptible to confounding factors such as alcohol consumption and obesity [8]. Although AMA has high specificity (≥95%), its titer does not correlate with disease severity or activity [9]. Furthermore, liver biopsy is invasive and associated with risks including hemorrhage and infection, making serial evaluations impractical [10]. A recent study demonstrated that more than 80% of AMA-positive patients with normal ALP levels exhibited typical PBC histopathology, highlighting a subset of underdiagnosed patients [11]. These limitations underscore the urgent need for novel, non-invasive, and accurate diagnostic biomarkers.

Urine represents an attractive source for biomarker discovery. It is obtainable non-invasively, can be collected repeatedly, and reflects the physiological and pathological states of multiple organ systems through plasma ultrafiltration [12]. Compared with plasma, urine contains fewer high-abundance proteins, making it more suitable for the detection of low-abundance proteins by mass spectrometry-based proteomics [13]. Urinary proteomics has been successfully applied to the discovery of biomarkers in kidney diseases [14], autoimmune conditions [15], and various liver diseases including hepatocellular carcinoma (HCC) and metabolic-associated fatty liver disease (MAFLD) [16,17]. However, studies on urinary proteomics in PBC remain scarce.

In this study, we performed label-free quantitative urinary proteomics coupled with bioinformatic analyses to identify differentially expressed urinary proteins in PBC patients, followed by ELISA-based validation of candidate biomarkers.

## 2. Results

### 2.1. Baseline Characteristics

The PBC cohort consisted of 9 patients (8 females) with a mean age of 51.33 ± 11.39 years. Their baseline liver biochemistries were as follows: alkaline phosphatase (ALP) of 361.67 ± 240.00 U/L, gamma-glutamyl transferase (GGT) of 460.22 ± 354.11 U/L, alanine transaminase (ALT) of 77.33 ± 62.37 U/L, aspartate transaminase (AST) of 90.33 ± 57.98 U/L, and total bilirubin (TB) of 15.34 ± 4.91 μmol/L; serum IgM averaged 4973.33 ± 3395.99 mg/L. The control group included 7 healthy individuals (5 females) with a mean age of 60.00 ± 4.32 years, all of whom exhibited normal liver function profiles. No intergroup differences were detected regarding sex distribution, age, total bile acid (TBA), albumin (ALB), or serum creatinine (Cr) (*p* ≥ 0.05). By contrast, significant disparities were identified in ALP, GGT, TB, direct bilirubin (DB), ALT, AST, IgM, and IgG levels between patients and healthy controls (*p* < 0.05), as summarized in Table 1.

In the ELISA validation cohort, 30 PBC patients (26 female; mean age 53.09 ± 10.33 years) and 20 healthy controls (14 female; mean age 50.20 ± 12.78 years) were included. Significant differences between groups were observed in biliary enzymes, bilirubin, and immunoglobulins (*p* < 0.001), whereas sex, age, and Cr were comparable (*p* ≥ 0.05) (Supplementary Table S1).

### 2.2. Identification of Differentially Expressed Urinary Proteins

**Figure 1:**
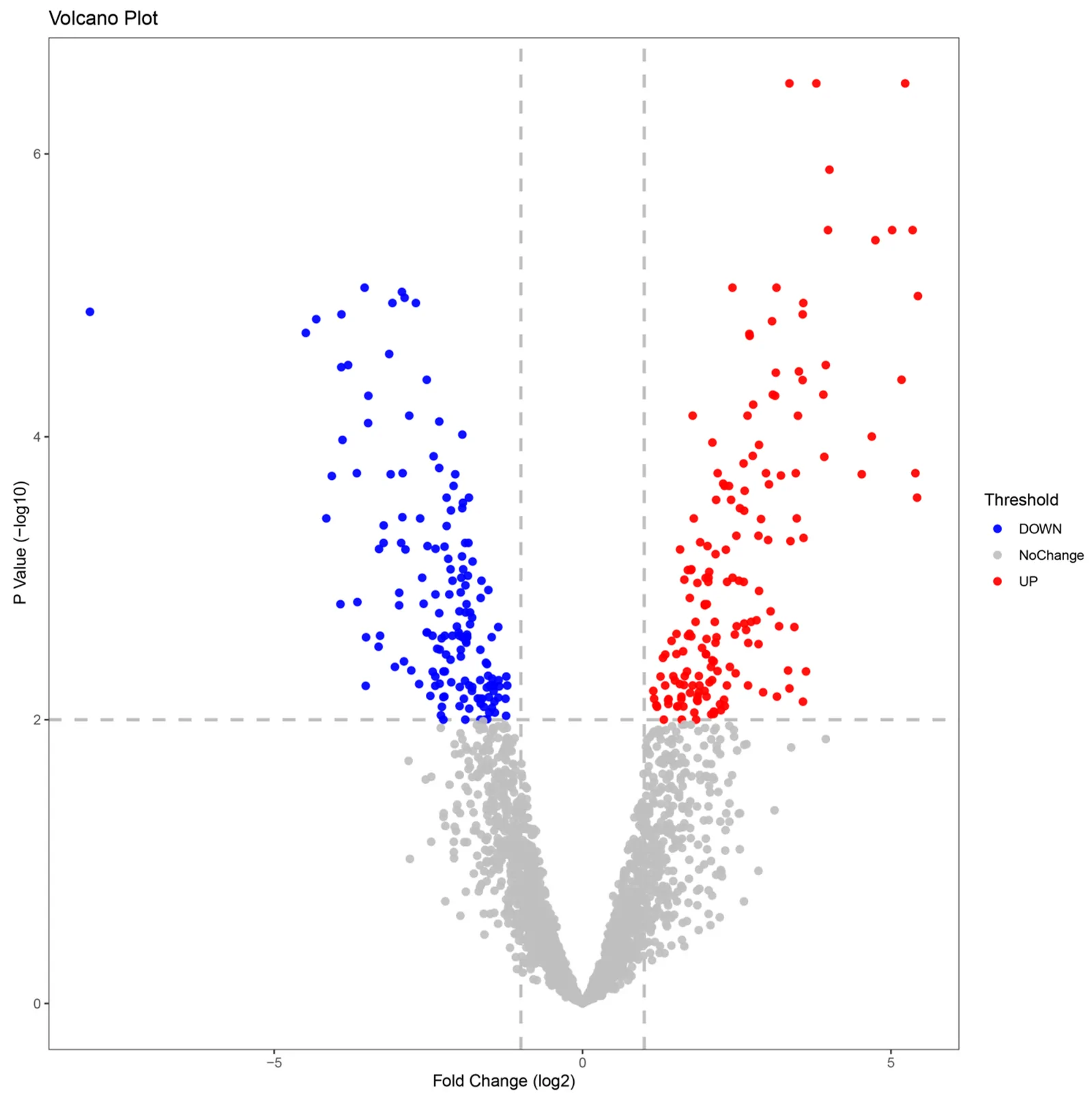
The volcano plot of differentially expressed urinary proteins.

After quality filtering and normalization (Supplementary Figures S1–S3), PCA demonstrated good intra-group reproducibility and clear inter-group separation (Supplementary Figure S4). With a cutoff of |FC| ≥ 2 and FDR < 0.01, a total of 194 differentially expressed urinary proteins were identified. A total of 109 were upregulated and 85 downregulated in PBC patients relative to healthy controls. The top 50 most significantly upregulated proteins included OPN (FC = 3.98), RAMP3 (FC = 3.94), CD44 (FC = 3.90), CXCL12 (FC = 3.43), CD74 (FC = 3.35), S100A8 (FC = 3.15), LAMP3 (FC = 3.08), PIK3CD (FC = 3.01), ACVR1B (FC = 3.62), and CCN4 (FC = 3.35), among others (Figure 1). Full lists of the top 50 upregulated and top 50 downregulated DEPs are provided in Supplementary Tables S2 and S3, respectively. The complete list of all differentially expressed urinary proteins were displayed in Supplementary Table S6.

### 2.3. GO and KEGG Enrichment Analysis

**Figure 2:**
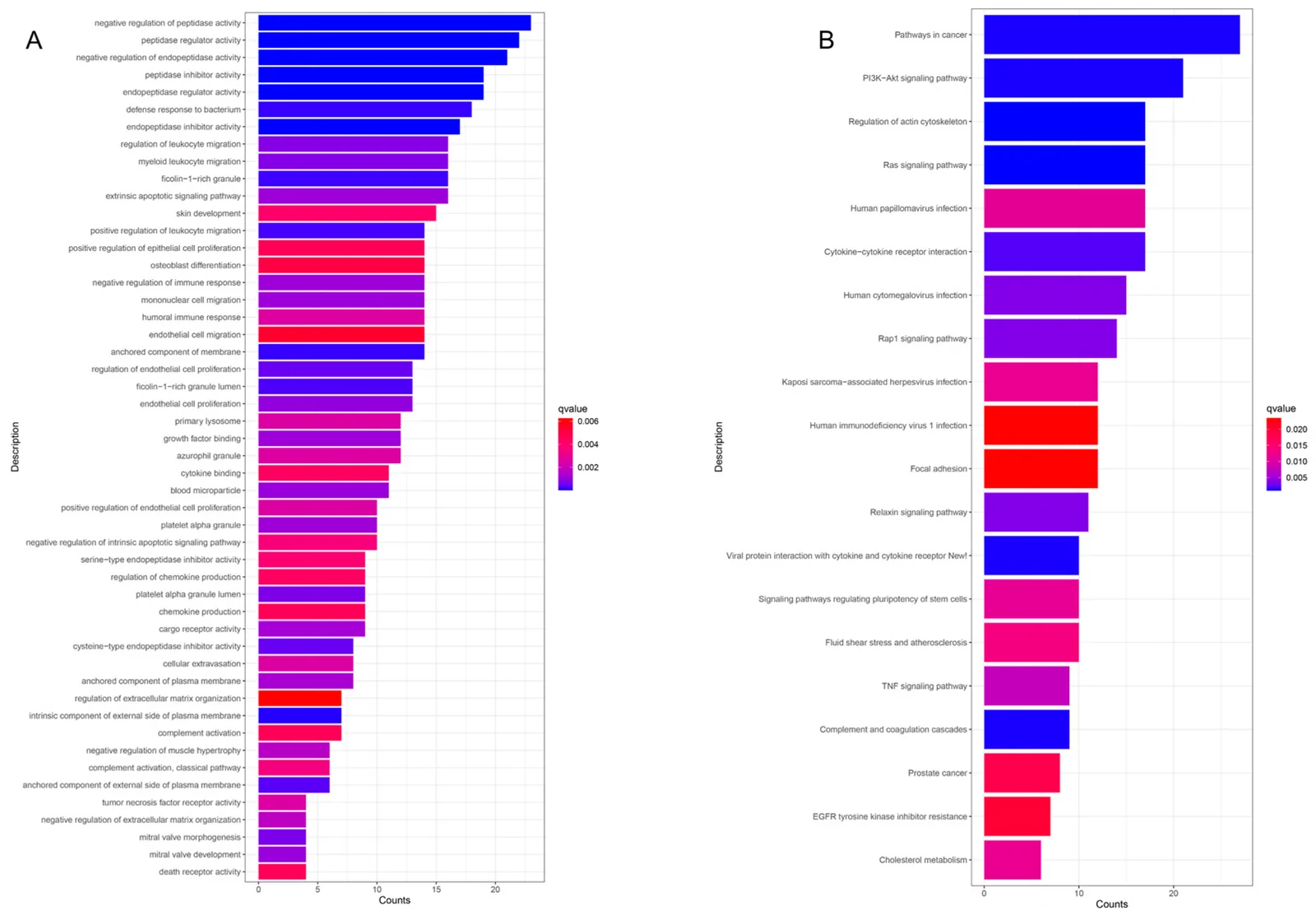
GO and KEGG enrichment analysis of the top 50 upregulated proteins in PBC patients. (**A**), GO analysis; (**B**), KEGG analysis.

GO analysis of the top 50 upregulated proteins revealed enrichment in biological processes including immune response, regulation of immune response, innate immune response, inflammatory response, complement activation (classical pathway), B cell receptor signaling pathway, Fc-gamma receptor signaling pathway involved in phagocytosis, and signal transduction. Molecular functions encompassed antigen binding, kinase activity, calcium ion binding, and signaling receptor binding. Cellular components were predominantly associated with extracellular exosomes, plasma membrane, and extracellular space (Figure 2A).

KEGG pathway analysis identified enrichment in PI3K-Akt signaling, Toll-like receptor (TLR) signaling, chemokine signaling, Wnt signaling, Notch signaling, leukocyte transendothelial migration, and ECM-receptor interaction pathways (Figure 2B).

Based on the convergence of GO immune-related categories and KEGG pathway enrichment, 10 candidate immune-related proteins were selected: OPN, RAMP3, CD44, ACVR1B, CXCL12, CD74, CCN4, S100A8, LAMP3, and PIK3CD.

### 2.4. Correlation Analysis with Clinical Parameters

Pearson correlation analysis of the 10 selected immune-related proteins with clinical biochemical indices revealed that three proteins showed the most consistent and statistically significant correlations (Table 2).

OPN was significantly correlated with ALT (r = 0.506, *p* = 0.040), AST (r = 0.525, *p* = 0.037), ALP (r = 0.543, *p* = 0.030), GGT (r = 0.549, *p* = 0.028), IgM (r = 0.700, *p* = 0.003), IgG (r = 0.634, *p* = 0.008), and AMA-M2 (r = 0.499, *p* = 0.049).

RAMP3 showed significant correlations with AST (r = 0.576, *p* = 0.020), ALP (r = 0.633, *p* = 0.009), GGT (r = 0.586, *p* = 0.017), TBA (r = 0.503, *p* = 0.047), IgM (r = 0.661, *p* = 0.005), and IgG (r = 0.640, *p* = 0.008).

**Figure 3:**
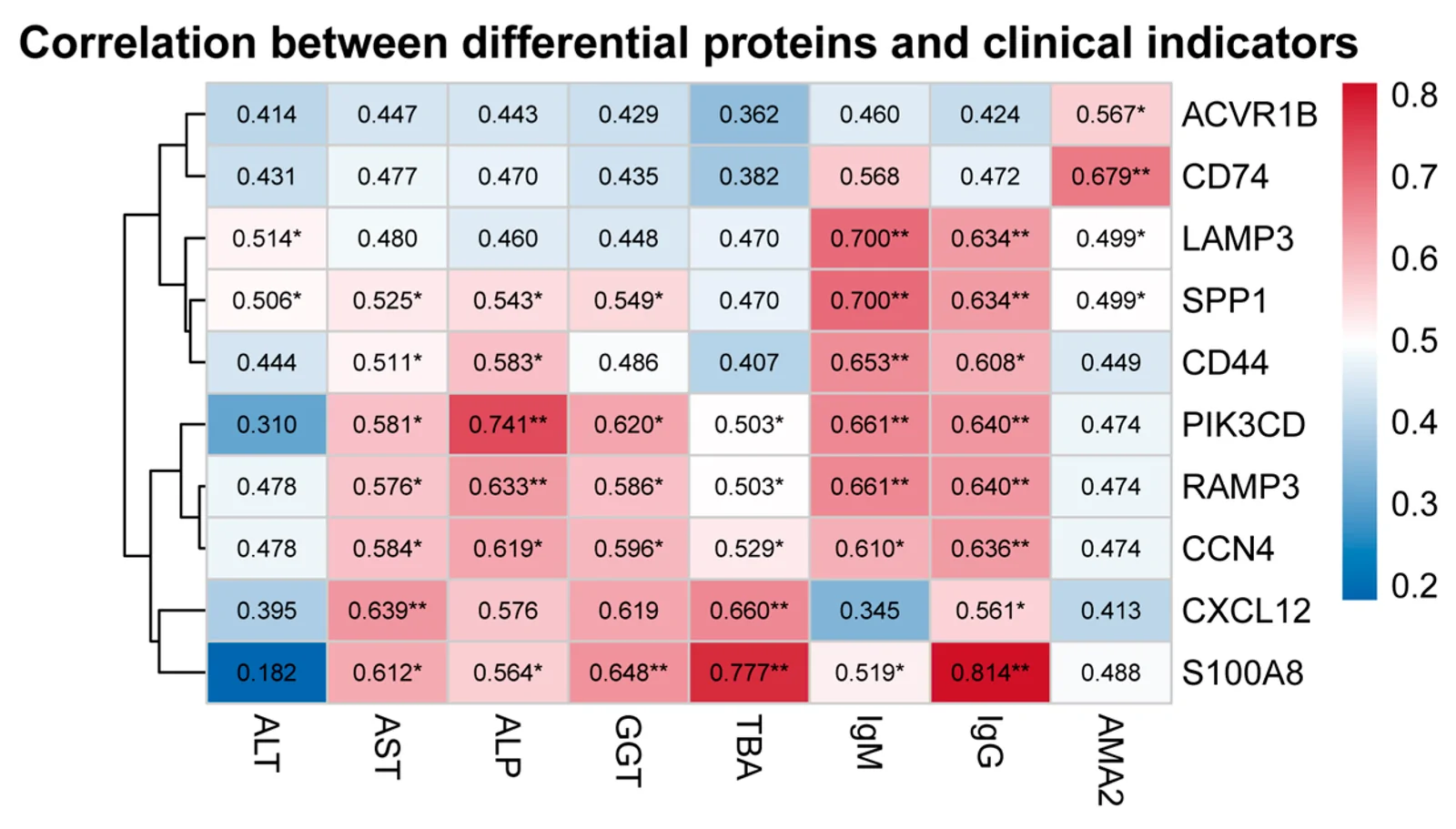
Correlation between differential proteins and clinical indicators. * *p* < 0.05, ** *p* < 0.01.

S100A8 demonstrated strong correlations with AST (r = 0.612, *p* = 0.012), ALP (r = 0.564, *p* = 0.023), GGT (r = 0.648, *p* = 0.007), TBA (r = 0.777, *p* < 0.001), IgM (r = 0.519, *p* = 0.039), and IgG (r = 0.814, *p* < 0.001). These exploratory data suggest potential associations between OPN, RAMP3, and S100A8 and established PBC disease activity markers in this small discovery cohort; however, these findings require further validation in larger cohorts (Figure 3).

Given the exploratory nature of this analysis in a relatively small cohort, both Pearson and Spearman correlation methods were applied, and consistent results were observed. Here we present the Pearson correlation results (Spearman results are provided in Supplementary Table S5).

### 2.5. The Result of ELISA Validation

**Figure 4:**
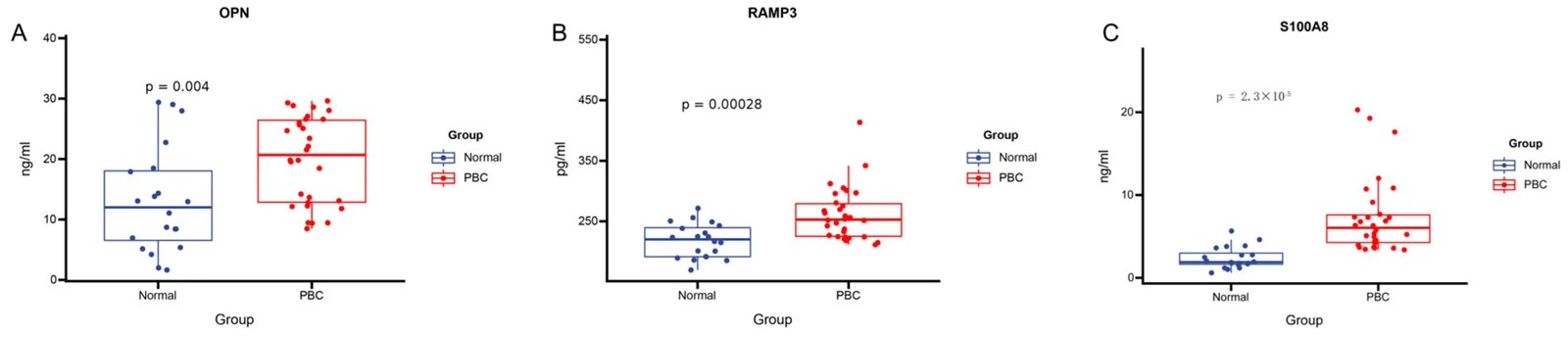
The ELISA results of three proteins in PBC patients compared with healthy controls. (**A**), OPN; (**B**), RAMP3; (**C**), S100A8.

ELISA confirmed significantly elevated urinary concentrations of all three proteins in PBC patients compared with healthy controls (all *p* < 0.01). OPN levels were 19.92 ± 7.19 ng/mL in PBC vs. 13.08 ± 8.74 ng/mL in controls. RAMP3 levels were 261.47 ± 44.02 pg/mL in PBC vs. 217.95 ± 28.03 pg/mL in controls. S100A8 levels were 7.35 ± 4.50 ng/mL in PBC vs. 2.39 ± 1.30 ng/mL in controls (Figure 4A–C). Standard curves for ELISA quantification were displayed in Supplementary Figure S5. The directional changes in protein expression were consistent with the mass spectrometry results, confirming the reliability of the proteomics data.

To evaluate the diagnostic performance of the three candidate biomarkers, we performed receiver operating characteristic (ROC) curve analysis. OPN and RAMP3 demonstrated excellent discriminative ability with AUC values of 1.00 (95% CI: 1.00–1.00), while S100A8 showed good diagnostic performance with an AUC of 0.89 (95% CI: 0.73–1.00). The combined three-protein model achieved an AUC of 1.00. AUC estimates may be optimistic and potentially affected by overfitting due to the small cohort size. Detailed ROC curves and full diagnostic performance metrics are provided in Supplementary Figure S6 and Supplementary Table S4.

## 3. Discussion

PBC is an immune-mediated chronic cholestatic liver disease whose rising global prevalence demands improved early and non-invasive diagnostic strategies [3,4]. The limitations of existing diagnostic tools, including the invasiveness of liver biopsy, the lack of AMA in approximately 5% of patients, and the non-specificity of liver enzymes, highlight the clinical need for novel biomarkers [7,8,9,10,11]. In this study, we employed urinary proteomics to identify and validate three candidate biomarkers. OPN, RAMP3, and S100A8 are significantly upregulated in PBC urine and correlated with disease activity indicators.

Urinary proteomics as a biomarker discovery platform: Urine is an excellent matrix for proteomics studies due to its non-invasive collection, stability of proteins, and reduced abundance of high-abundance interfering proteins compared with plasma [13,18]. Recent studies have demonstrated the utility of urinary proteomics in liver diseases, including the identification of noninvasive diagnostic peptides for liver fibrosis [19] and HCC [16], supporting the validity of this approach in PBC.

Osteopontin (OPN) is a multifunctional glycoprotein encoded by the SPP1 gene, implicated in inflammation, fibrosis, angiogenesis, and immune modulation [20]. OPN is minimally expressed in normal liver but is upregulated in biliary epithelial cells (BECs), Kupffer cells, and hepatic stellate cells under pathological conditions [21]. Its interaction with integrin receptors promotes cell migration, adhesion, and pro-inflammatory signaling. OPN has been identified as a biomarker for liver fibrosis in multiple etiologies [22,23] and as a prognostic marker in HCC [24]. In our study, OPN was the second most significantly upregulated protein and was strongly correlated with ALP, GGT, IgM, and AMA-M2, suggesting its involvement in PBC-associated biliary damage and immune dysregulation. KEGG analysis indicated that OPN participates in TLR signaling pathways, which are known to mediate BEC apoptosis in autoimmune cholangitis models [25], providing a mechanistic rationale for its elevation in PBC urine.

Receptor Activity-Modifying Protein 3 is a type I transmembrane protein that modulates the function of G protein-coupled receptors (GPCRs), including the calcitonin receptor-like receptor (CRLR) and G protein-coupled estrogen receptor 1 (GPER1) [26]. The CRLR/RAMP3 complex serves as a high-affinity receptor for adrenomedullin (AM), a vasoactive peptide with roles in cardiovascular homeostasis, angiogenesis, and inflammation [27]. Genetic analyses have demonstrated that AM and RAMP3 are directly regulated by estrogen receptor signaling [28], and GPER1-RAMP3 interactions have been reported to confer sex-dependent cardiovascular protection [29]. Given that PBC disproportionately affects women and is associated with hormonal influences, the upregulation of RAMP3 in PBC urine may reflect estrogen-mediated dysregulation of GPCR signaling. Furthermore, bile acids are established activators of GPCR signaling, and their metabolic dysregulation in cholestatic disease may further modulate RAMP3 expression [30]. These observations warrant further mechanistic investigation.

S100A8 is a calcium and zinc binding protein and is primarily expressed in phagocytes and classified as a damage-associated molecular pattern (DAMP) molecule [31]. S100A8 forms a heterodimer with S100A9, which functions as an endogenous ligand for TLR4 and RAGE, thereby amplifying inflammatory signaling [32,33]. In autoimmune diseases, S100A8/A9 is markedly elevated and serves as a disease activity biomarker [34,35]. Notably, S100A9 has been identified as a differentially expressed biliary protein in primary sclerosing cholangitis (PSC) [36], a related cholestatic liver disease. In our study, S100A8 showed the strongest correlation with TBA and IgG among the three candidate proteins, and KEGG analysis implicated it in the IL-17 signaling pathway, which is dysregulated in multiple autoimmune conditions [37]. These findings collectively support a role for S100A8 in PBC-associated innate immune activation and position it as a promising urinary biomarker.

Regarding biomarker specificity, it should be noted that OPN and S100A8 are well-characterized inflammation-associated molecules that may be elevated in various inflammatory conditions. However, the strong correlations observed between all three urinary proteins and cholestasis-specific biochemical indices (ALP, GGT, TBA) suggest that their elevation is closely associated with biliary epithelial injury rather than non-specific systemic inflammation. In particular, RAMP3, which has been less extensively studied in liver diseases, may represent a more PBC-specific candidate. Future studies including disease control groups such as primary sclerosing cholangitis (PSC) and autoimmune hepatitis (AIH) are needed to fully establish the diagnostic specificity of these urinary biomarkers.

Several limitations of this study should be acknowledged. First, the proteomics discovery cohort was relatively small, which may have limited statistical power and introduced sampling bias. However, we subsequently validated the three candidate biomarkers by ELISA in a larger cohort (30 PBC patients and 20 healthy controls), which partially mitigates this concern. Nevertheless, our findings remain exploratory and require further validation in larger multicenter prospective cohorts. Second, the diagnostic specificity of OPN, RAMP3, and S100A8 has not been evaluated against other cholestatic or autoimmune liver conditions. Third, the mechanistic basis for the urinary elevation of these proteins in PBC remains to be fully elucidated. Future studies incorporating artificial intelligence and machine learning algorithms to integrate multi-omics data with clinical parameters may further improve diagnostic performance and enable personalized risk stratification for PBC [38]. Fourth, the diagnostic performance (AUC) estimated in this relatively small cohort may be overestimated due to overfitting, and future large-scale validation studies are required to determine the true diagnostic accuracy of these biomarkers. In addition, the correlation analyses were performed in the small discovery cohort, and although Spearman correlation and FDR correction were applied as sensitivity analyses, these findings remain exploratory and require confirmation in larger cohorts. Furthermore, detailed information on disease stage and long-term treatment response was limited in this cohort.

## 4. Materials and Methods

### 4.1. Study Participants

This study prospectively enrolled PBC patients and healthy controls between January 2022 and December 2022 at West China Hospital, Sichuan University. PBC diagnosis was established according to the 2018 American Association for the Study of Liver Diseases (AASLD) Practice Guidance [7]. Inclusion criteria were: (1) fulfillment of PBC diagnostic criteria; (2) liver biopsy histology consistent with PBC; (3) complete clinical and laboratory data. Exclusion criteria included: (1) co-existing autoimmune hepatitis, primary sclerosing cholangitis, or other non-immune hepatic diseases (drug-induced hepatitis, alcoholic liver disease, or hereditary metabolic liver disease); (2) secondary liver damage due to other major organ dysfunction; (3) severe renal insufficiency or massive proteinuria.

For the proteomics discovery cohort, 9 biopsy-confirmed PBC patients and 7 healthy controls with complete clinical data were selected. For the ELISA validation cohort, 30 PBC patients and 20 healthy controls were enrolled. All PBC patients were receiving ursodeoxycholic acid (UDCA) as first-line therapy at the time of sample collection. Written informed consent was obtained from all participants. The study was approved by the Institutional Review Board of West China Hospital and conducted in accordance with the Declaration of Helsinki.

### 4.2. Sample Collection and Processing

First-morning midstream urine specimens were collected from all subjects without any artificial dilution on the day of admission or clinic visit. Uniform collection instructions were issued to all participants to standardize water intake and fasting status 12 h before sampling, which reduced potential intergroup variability in urine dilution degree. Normalization by urinary creatinine (mg/g Cr) was not adopted in this study, and all quantitative results of urinary proteins were presented as original concentrations (ng/mL) without creatinine adjustment to avoid artificial deviation introduced by renal function interference. Pearson correlation analysis further confirmed no significant association between urinary creatinine and the concentrations of OPN, RAMP3 or S100A8 (all *p* > 0.05), indicating that urine dilution exerted negligible confounding effects on these biliary-derived proteins. Samples were centrifuged at 3000 rpm for 10 min, and supernatants were aliquoted and stored at −80 °C until analysis. For proteomics, urine proteins were extracted by ultracentrifugation precipitation. Briefly, samples were reduced with 20 mM dithiothreitol (DTT) at 37 °C for 4 h, alkylated with 50 mM iodoacetamide (IAA) at 37 °C for 30 min, and digested with trypsin (Promega, Madison, WI, USA) and Lys-C at 37 °C for 16 h using filter-aided sample preparation (FASP). Peptides were desalted using C18 spin columns and dried by lyophilization.

### 4.3. HPLC-ESI-IT/MS Analysis

Dried peptides were resuspended in mobile phase A (0.1% formic acid in water) and separated on a capillary column (C18, 1.9 μm, 0.15 × 120 mm) using an Orbitrap Exploris 480 mass spectrometer coupled with an EASY-nLC 1200 system (Thermo Fisher Scientific, Waltham, MA, USA). The elution gradient was as follows: 10 min at 6% B → 15 min at 9–14% B → 50 min at 14–30% B → 30 min at 30–40% B → 3 min at 40–95% B → 7 min at 95% B → 1 min at 95–6% B → 4 min at 6% B, at a flow rate of 600 nL/min.

### 4.4. Data Analysis and Bioinformatics

Urinary proteins extracted from PBC patients and healthy controls were subjected to proteomic profiling. Raw qualitative and quantitative protein data were acquired using Spectronaut version 14.8 database search software. Missing value distributions across individual samples and each protein were calculated for quality control. Raw data were median-normalized and log_2_-transformed sequentially. Proteins with missing values exceeding 50% were filtered out, and the remaining missing entries were imputed. Coefficients of variation (CV) of all proteins were then computed, and proteins with CV > 30% were excluded to eliminate highly variable signals.

Differential protein expression was assessed using the Limma R package, with *p* values corrected by the Benjamini–Hochberg (BH) method (false discovery rate, FDR). Proteins with |fold change (FC)| ≥ 2 and FDR < 0.01 were considered significantly differentially expressed. Principal component analysis (PCA) and volcano plots were generated using R statistical software (version 4.2.1). GO functional enrichment and KEGG pathway analyses were performed to annotate the top 50 upregulated proteins using the DAVID Bioinformatics Resources (https://davidbioinformatics.nih.gov/). Pearson correlation analysis was used to evaluate associations between candidate immune-related proteins and clinical biochemical parameters.

### 4.5. ELISA Validation

Urinary levels of candidate proteins were quantified by ELISA in the validation cohort using commercially available kits (Elabscience Biotechnology, Wuhan, China). All assays were performed according to manufacturers’ instructions. Optical density was measured at 450 nm. Protein concentrations were calculated from standard curves. All ELISA assays were performed by laboratory personnel blinded to disease status, and each sample was analyzed in technical duplicates, with the mean concentration used for statistical analysis.

### 4.6. Statistical Analysis

Statistical analyses were performed using SPSS 27.0. Normally distributed continuous variables are expressed as mean ± standard deviation (SD), and non-normally distributed variables as median (interquartile range). Categorical variables are expressed as frequency or percentage. Between-group comparisons were performed using Student’s *t*-test or Mann–Whitney U test as appropriate. Correlation analysis was performed using Pearson’s method. As a sensitivity analysis, Spearman rank correlation was also conducted, and the results were consistent with Pearson correlation. Benjamini–Hochberg false discovery rate (FDR) correction was applied to adjust for multiple testing. Graphs were generated using GraphPad Prism 8.0. *p* < 0.05 was considered statistically significant.

## 5. Conclusions

This exploratory study demonstrates that urinary proteomics can identify novel diagnostic candidates in PBC. OPN, RAMP3, and S100A8 are significantly upregulated in the urine of PBC patients, are correlated with established clinical disease activity markers, and are confirmed by ELISA in a validation cohort. These three proteins hold promise as candidate non-invasive urinary candidate biomarkers for PBC; however, they are not yet established diagnostic biomarkers, and their specificity against other cholestatic and autoimmune liver diseases has not been evaluated in this study. Future larger, multicenter prospective studies including disease control groups are warranted to fully validate their diagnostic specificity, accuracy, and clinical utility.

## Figures and Tables

**Table 1:** Baseline characteristics of PBC patients and healthy controls in the urinary proteomics cohort.

Characteristics	PBC (N = 9)	Control (N = 7)	*p*
Gender, %			
Male	1 (11.1%)	2 (28.6%)	0.375
Female	8 (88.9%)	5 (71.4%)	
Age/year	51.33 ± 11.39	60.00 ± 4.32	0.19
ALP/(U/L)	361.67 ± 240.00	58.57 ± 9.71	<0.001
GGT/(U/L)	460.22 ± 354.11	19.70 ± 10.08	<0.001
TB/(μmol/L)	15.34 ± 4.91	6.64 ± 1.04	<0.001
DB/(μmol/L)	7.46 ± 3.60	3.03 ± 0.62	0.006
TBA/(μmol/L)	26.12 ± 24.48	2.77 ± 1.85	0.055
ALT/(U/L)	77.33 ± 62.37	19.13 ± 9.66	0.023
AST/(U/L)	90.33 ± 57.98	23.00 ± 6.85	0.008
ALB/(g/L)	46.74 ± 15.77	46.67 ± 1.55	0.210
Cr/(μmol/L)	58.89 ± 10.42	62.14 ± 6.62	0.484
IgM/(mg/L)	4973.33 ± 3395.99	807.14 ± 48.21	<0.001
IgG/(g/L)	18.11 ± 6.83	9.39 ± 1.25	<0.005

Footer: ALP alkaline phosphatase, GGT gamma-glutamyl transferase, TB total bilirubin, DB direct bilirubin, TBA total bile acid, ALT alanine aminotransferase, AST aspartate aminotransferase, ALB albumin, Cr serum creatinine, IgM immunoglobulin M, IgG immunoglobulin G, and PBC primary biliary cholangitis.

**Table 2:** Correlation analysis between differential proteins and clinical indicators.

Uniprot	Gene Name	ALT, r *p*	AST, r *p*	ALP, r *p*	GGT, r *p*	TBA, r *p*	IgM, r *p*	IgG, r *p*	AMA2, r *p*
P10451	***OPN***	**0.506, 0.040**	**0.525, 0.037**	**0.543, 0.030**	**0.549, 0.028**	0.47, 0.066	**0.700, 0.003**	**0.634, 0.008**	0.499, 0.049
O60896	***RAMP3***	0.478, 0.060	**0.576, 0.020**	**0.633, 0.009**	**0.586, 0.017**	**0.503, 0.047**	**0.661, 0.005**	**0.64, 0.008**	0.474, 0.064
P16070	*CD44*	0.444, 0.085	**0.511, 0.043**	**0.583, 0.018**	0.486, 0.560	0.407, 0.118	**0.653, 0.006**	**0.608, 0.012**	0.449, 0.081
P36896	*ACVR1B*	0.414, 0.111	0.447, 0.083	0.443, 0.086	0.429, 0.097	0.362, 0.169	0.460, 0.073	0.424, 0.102	0.567, 0.022
P48061	*CXCL12*	0.395, 0.130	**0.639, 0.008**	**0.576, 0.200**	**0.619, 0.110**	**0.660, 0.005**	0.345, 0.191	**0.561, 0.024**	0.413, 0.112
P04233	*CD74*	0.431, 0.095	0.477, 0.062	0.470, 0.066	0.435, 0.092	0.382, 0.144	**0.568, 0.220**	0.472, 0.065	**0.679, 0.004**
O95388	*CCN4*	0.478, 0.061	**0.584, 0.017**	**0.619, 0.011**	**0.596, 0.015**	**0.529, 0.035**	**0.610, 0.012**	**0.636, 0.008**	0.474, 0.064
P05109	***S100A8***	0.182, 0.501	**0.612, 0.012**	**0.564, 0.023**	**0.648, 0.007**	**0.777, <0.001**	**0.519, 0.039**	**0.814, <0.001**	0.488, 0.055
Q9UQV4	*LAMP3*	**0.514, 0.041**	0.480, 0.060	0.460, 0.073	0.448, 0.082	0.470, 0.066	**0.700, 0.003**	**0.634, 0.008**	0.499, 0.049
O00329	*PIK3CD*	0.310, 0.243	**0.581, 0.018**	**0.741, 0.001**	**0.620, 0.010**	**0.503, 0.047**	**0.661, 0.005**	**0.640, 0.008**	0.474, 0.064

Note: ALP alkaline phosphatase, GGT gamma-glutamyl transferase, TBA total bile acid, ALT alanine aminotransferase, AST aspartate aminotransferase, IgM immunoglobulin M, and IgG immunoglobulin G. ‘r’ stands for correlation coefficient. The bold formatting were used to indicate statistically significant correlations (*p* < 0.05).

## Data Availability

The original contributions presented in this study are included in the article/Supplementary Materials. Further inquiries can be directed to the corresponding authors.

## Supplementary Materials

The following supporting information can be downloaded at: https://www.mdpi.com/article/10.3390/ijms27177584/s1.

## Abbreviations

The following abbreviations are used in this manuscript:

PBC	Primary biliary cholangitis
ALT	Alanine aminotransferase
AST	Aspartate aminotransferase
ALP	Alkaline phosphatase
GGT	Gamma-glutamyl transferase
TBA	Total bile acid
TBIL	Total bilirubin
ALB	Albumin
IgM	Immunoglobulin M
IgG	Immunoglobulin G
AMA-M2	Anti-mitochondrial Antibody type 2
ANA	Antinuclear antibody
gp210	Anti-glycoprotein 210 antibody
Sp100	Anti-nucleoprotein 100 antibody
UDCA	Ursodeoxycholic Acid
PSC	Primary sclerosing cholangitis
MAFLD	Metabolic-associated fatty liver disease
LC-MS	Liquid chromatograph mass spectrometer
ESI-IT-MS	Electrospray ionization ion trap mass spectrometry
DTT	Dithiothreitol
IAA	Iodoacetamide
GO	Gene Ontology
KEGG	Kyoto Encyclopedia of Genes and Genomes
FDR	False discovery rate
FC	Fold Change
ELISA	Enzyme-linked immunosorbent assay
OPN	Osteopontin
SPP1	Secreted phosphoprotein 1
BECs	Biliary epithelial cells
GPCR	G protein-coupled receptor
