# Supplementary material for: Label-Free Urinary Proteomics Uncovers Immune-Related Non-Invasive Biomarkers for Primary Biliary Cholangitis
Source: Int J Mol Sci. 2026 Aug 24;27(17):7584. doi: 10.3390/ijms27177584 (PMC13566571; doi:10.3390/ijms27177584)
Supplement: Supplementary file 1 [file ijms-27-07584-s001.zip › Supplementary caption.pdf]

International Journal of
Molecular Sciences

MDPI

Supplementary Materials.

Supplementary Table S1 Baseline characteristics of subjects in ELISA validation cohort.

Supplementary Table S2 Top 50 Upregulated differentially expressed proteins in urine samples of PBC patients.

Supplementary Table S3 Top 50 Downregulated differentially expressed proteins in urine samples of PBC patients.

Supplementary Figure S1 Missing value status of urinary proteins in each sample during proteomic profiling analysis. OK: Samples with missing values less than 30%, indicating good sample quality. Bad: Samples with missing values exceeding 30%, indicating poor sample quality.

Supplementary Figure S2 Missing value distribution of each protein across all samples. Missing: The proportion of undetected signals of a protein in all samples ≥ 27%. Observed: The proportion of detectable signals of a protein in all samples ≥ 73%.

Supplementary Figure S3 Coefficient of variation (CV) distribution of all urinary proteins after missing value filtering and imputation; proteins with CV > 30% were excluded.

Supplementary Figure S4 Principal component analysis of urinary proteins in patients with PBC and healthy controls.

Supplementary Figure S5 Standard curve for ELISA quantification. A, OPN; B, RAMP3; C, S100A8.

Supplementary Figure S6: ROC curves of OPN, RAMP3, S100A8 and the combined three-protein model for PBC diagnosis.

Supplementary Table S4: Diagnostic performance of urinary OPN, RAMP3, S100A8 and the combined model (AUC, sensitivity, specificity, PPV, NPV, accuracy).

Supplementary Table S5. Spearman rank correlation analysis between candidate urinary proteins and clinical biochemical indicators in the proteomics discovery cohort.

Supplementary Table S6. Complete list of differentially expressed urinary proteins between PBC patients and healthy controls.
