# Supplementary material for: Label-Free Urinary Proteomics Uncovers Immune-Related Non-Invasive Biomarkers for Primary Biliary Cholangitis
Source: Int J Mol Sci. 2026 Aug 24;27(17):7584. doi: 10.3390/ijms27177584 (PMC13566571; doi:10.3390/ijms27177584)
Supplement: Supplementary file 1 [file ijms-27-07584-s001.zip › Supplementary Table S1.pdf]

Baseline Characteristics of subjects in ELISA validation cohort

Characteristics	PBC (N=30)	Controls(N=20)	*P*
Gender, %			
Male	4 (13.3%)	6 (30%)	0.149
Female	26 (86.7%)	14 (70%)	
Age/year	53.09±10.33	50.20±12.78	0.382
ALP(U /L)	258.27±209.65	59.75±7.52	＜0.001
GGT(U/L)	153.00(59.25,495.50)	18.40(14.80,24.70)	＜0.001
TB(μmol/L)	17.85(12.00,25.53)	6.1(5.80,7.53)	＜0.001
DB(μmol/L)	7.15(3.70,12.53)	2.95(2.53,3.78)	＜0.001
TBA(μmol/L)	46.47±62.31	3.59±1.72	＜0.001
ALT(U /L)	45.00(25.00,86.75)	14.80(11.98,21.13)	＜0.001
AST(U /L)	52.00(33.50,117.00)	19.95(17.35,24.98)	＜0.001
ALB（g/L）	42.83±10.42	45.34±2.66	0.032
Cr(μmol/L)	91.34±154.62	63.95±6.27	0.250
IgM（mg/L）	3589.53±2356.64	822.50±59.37	＜0.001
IgG（g/L）	16.25±5.97	9.92±1.38	＜0.001

Note: ALP alkaline phosphatase, GGT gamma-glutamyl transferase, TB total bilirubin, DB direct bilirubin, TBA total bile acid, ALT alanine aminotransferase, AST aspartate aminotransferase, ALB albumin, Cr serum creatinine, IgM immunoglobulin M, and IgG immunoglobulin G, PBC primary biliary cholangitis.
