# Supplementary material for: Label-Free Urinary Proteomics Uncovers Immune-Related Non-Invasive Biomarkers for Primary Biliary Cholangitis
Source: Int J Mol Sci. 2026 Aug 24;27(17):7584. doi: 10.3390/ijms27177584 (PMC13566571; doi:10.3390/ijms27177584)
Supplement: Supplementary file 1 [file ijms-27-07584-s001.zip › Supplementary Table S2.pdf]

Top 50 Upregulated differentially expressed proteins in urine samples of PBC patients

UNIPROT	Gene Name	FC	*P*	FDR
Q9NQX4	MYO5C	5.4366242	4.67E-08	1.01E-05
P06331	NA	5.42316839	7.16E-06	0.000269254
P32320	CDA	5.39596102	3.89E-06	0.000180748
A0A0B4J1U7	NA	5.351035758	6.53E-09	3.46E-06
Q14508	WFDC2	5.230067713	2.96E-10	3.17E-07
P01597	NA	5.171455121	4.49E-07	3.95E-05
P63261	ACTG1	5.019042304	8.59E-09	3.46E-06
P16403	H1-2	4.747480898	1.16E-08	4.08E-06
P62820	RAB1A	4.688842279	1.66E-06	9.96E-05
Q96GN5	CDCA7L	4.525876373	4.16E-06	0.000183909
P15328	FOLR1	4.002433796	1.84E-09	1.29E-06
P10451	SPP1	3.978966986	8.38E-09	3.46E-06
O60896	RAMP3	3.943055762	3.09E-07	3.11E-05
O60234	GMFG	3.918883015	2.61E-06	0.000138634
P16070	CD44	3.903748147	6.43E-07	5.03E-05
P02760	AMBP	3.790246718	3.38E-10	3.17E-07
P36896	ACVR1B	3.622733721	0.00036239	0.004557348
Q9Y219	JAG2	3.582939057	1.71E-05	0.000518445
P02768	ALB	3.57871754	6.84E-08	1.13E-05
Q14166	TTLL12	3.573191525	0.00077253	0.007452754
P02765	AHSG	3.56880999	9.20E-08	1.36E-05
P01889	HLA-B	3.567385571	4.79E-07	3.97E-05
Q13618	CUL3	3.507634241	3.68E-07	3.45E-05
Q9BYD5	CNFN	3.491767101	1.08E-06	7.10E-05
P05090	APOD	3.472457102	1.14E-05	0.000377273
Q92765	FRZB	3.456784223	3.75E-06	0.000180748
P48061	CXCL12	3.433940331	0.00013006	0.002212163
Q9UEW8	STK39	3.370867368	1.84E-05	0.000546343

UNIPROT	Gene Name	FC	*P*	FDR
P04233	CD74	3.354216916	0.00055896	0.006009874
O95388	CCN4	3.354201661	2.73E-10	3.17E-07
Q8NHQ9	DDX55	3.332025716	0.00034893	0.004488229
Q13439	GOLGA4	3.215956348	4.33E-06	0.00018774
P06310	NA	3.185957377	0.00012582	0.002183535
P05109	S100A8	3.151272744	0.00066991	0.006862363
P01876	NA	3.144101686	2.96E-08	8.83E-06
P0DJD8	PGA3	3.133441429	3.88E-07	3.52E-05
P0DOY3	NA	3.121858346	6.80E-07	5.13E-05
Q9UQV4	LAMP3	3.084813869	6.40E-07	5.03E-05
P01834	NA	3.071925394	1.19E-07	1.52E-05
P02750	LRG1	3.048550953	9.18E-05	0.001712435
P01877	NA	3.022068527	5.23E-06	0.000216796
O00329	PIK3CD	3.008435835	1.79E-05	0.000536538
P01034	CST3	2.973876106	3.89E-06	0.000180748
Q8WWV6	FCAMR	2.927119851	0.00060635	0.00639738
P01601	NA	2.893665761	1.19E-05	0.000381537
P0DTE1	NA	2.861150744	5.89E-05	0.001228712
P49908	SELENOP	2.860594022	2.02E-06	0.000114065
P02144	MB	2.852810073	0.00020112	0.002920381
P15814	IGLL1	2.851807535	1.62E-05	0.000500739
P01699	NA	2.820907452	0.00010971	0.001981066

Note: FC, fold change; FDR, false discovery rate.
