# Supplementary material for: Label-Free Urinary Proteomics Uncovers Immune-Related Non-Invasive Biomarkers for Primary Biliary Cholangitis
Source: Int J Mol Sci. 2026 Aug 24;27(17):7584. doi: 10.3390/ijms27177584 (PMC13566571; doi:10.3390/ijms27177584)
Supplement: Supplementary file 1 [file ijms-27-07584-s001.zip › Supplementary Table S3.pdf]

Top 50 Downregulated differentially expressed proteins in urine samples of PBC patients

UNIPROT	Gene Name	FC	*P*	FDR
Q6UXB4	CLEC4G	-2.41486723	2.54E-06	0.000137352
P01303	NPY	-2.429595375	0.00036546	0.004575517
P21796	VDAC1	-2.431977198	0.00016263	0.002547692
P35527	KRT9	-2.467491023	0.00065007	0.006782426
P18428	LBP	-2.51373877	2.14E-05	0.00059203
P01040	CSTA	-2.519293115	0.0001457	0.002416292
P24530	EDNRB	-2.525458739	4.63E-07	3.95E-05
Q13162	PRDX4	-2.52651384	0.00014582	0.002416292
Q9NXU5	ARL15	-2.576349173	7.69E-05	0.001514152
Q9P2T1	GMPR2	-2.600474142	4.33E-05	0.000991266
O00571	DDX3X	-2.632970984	1.17E-05	0.000377666
Q9BQ61	TRIR	-2.649670167	0.00048448	0.005596522
P00742	F10	-2.701779365	6.77E-08	1.13E-05
P13804	ETFA	-2.776805143	0.00034691	0.00448283
Q92542	NCSTN	-2.809951531	1.03E-06	7.09E-05
P46531	NOTCH1	-2.87082354	2.37E-05	0.000625993
Q9UP38	FZD1	-2.884747312	5.17E-08	1.04E-05
P60903	S100A10	-2.895231604	0.00029099	0.003869932
Q68CJ9	CREB3L3	-2.916416617	3.91E-06	0.000180748
Q6NZI2	CAVIN1	-2.921030917	1.09E-05	0.000370069
Q9Y287	ITM2B	-2.928204836	4.03E-08	9.46E-06
P12277	CKB	-2.940601611	1.97E-05	0.000562637
Q86YZ3	HRNR	-2.97228893	6.16E-05	0.001266347
Q9NP84	TNFRSF12A	-2.973399508	8.21E-05	0.001553113
P02795	MT2A	-3.041849539	0.00032332	0.004230411
Q96FE5	LINGO1	-3.082311166	6.76E-08	1.13E-05
Q15517	CDSN	-3.110697277	4.18E-06	0.000183909
P05997	COL5A2	-3.134748014	2.40E-07	2.60E-05

UNIPROT	Gene Name	FC	*P*	FDR
Q9UGM3	DMBT1	-3.22412776	1.34E-05	0.000422944
P12838	DEFA4	-3.225078134	1.98E-05	0.000562637
Q9Y5X1	SNX9	-3.282757645	0.00016205	0.002547692
P35080	PFN2	-3.299590784	2.32E-05	0.000621344
P01599	NA	-3.306661873	0.00021089	0.003046502
P55259	GP2	-3.472870148	6.92E-07	5.13E-05
A0A1B0GUA5	NA	-3.476657521	1.28E-06	7.99E-05
Q9NQ76	MEPE	-3.510039202	0.00017156	0.002612396
Q6VY07	PACS1	-3.515440469	0.00052363	0.005759753
P20333	TNFRSF1B	-3.533022111	3.28E-08	8.83E-06
Q96MG2	JSRP1	-3.650049279	7.43E-05	0.001472976
Q9Y6R1	SLC4A4	-3.659355584	3.64E-06	0.000180748
P80370	DLK1	-3.800357174	3.03E-07	3.11E-05
P35442	THBS2	-3.891749722	1.79E-06	0.00010519
P38571	LIPA	-3.908604993	9.68E-08	1.36E-05
P02656	APOC3	-3.911507068	3.31E-07	3.22E-05
Q6UWH4	GASK1B	-3.924727718	7.85E-05	0.001523841
O95359	TACC2	-4.065106882	4.43E-06	0.000189281
P13521	SCG2	-4.154068548	1.13E-05	0.000377273
P02452	COL1A1	-4.317625488	1.10E-07	1.47E-05
A0A075B6J9	NA	-4.48683886	1.51E-07	1.84E-05
Q9BYE2	TMPRSS13	-7.986295017	8.35E-08	1.31E-05

Note: FC, fold change; FDR, false discovery rate.
